# REPLY BY THE AUTHORS: Re: Artificial urinary sphincter for urinary incontinence after radical prostatectomy: a historical cohort from 2004 to 2015

**DOI:** 10.1590/S1677-5538.IBJU.2017.0074.1

**Published:** 2017

**Authors:** Augusto Cesar Soares dos Santos, Luíza de Oliveira Rodrigues, Daniela Castelo Azevedo, Lélia Maria de Almeida Carvalho, Mariana Ribeiro Fernandes, Sandra de Oliveira Sapori Avelar, Maria da Glória Cruvinel Horta, Silvana Márcia Bruschi Kelles

**Affiliations:** 1 Grupo de Avaliação de Tecnologia em Saúde, Unimed BH, MG, Brasil;; 2 Núcleo de Avaliação de Tecnologia em Saúde, Hospital das Clínicas, Universidade Federal de Minas Gerais (UFMG), MG, Brasil

*To the editor,*

We appreciated the comments made on our article entitled “Re: Artificial urinary sphincter for urinary incontinence after radical prostatectomy: a historical cohort from 2004 to 2015” (1, 2) as we believe that discussions regarding the artificial urinary sphincter (AUS) in Brazil are scarce and should be encouraged. Other authors, aligned with our results and from different international locations, have recently also reported concern with the AUS because of its high revision and explantation rates due to mechanical failure, urethral atrophy, infection, erosion, etc (3, 4).

A report of a Consensus Conference (5), based on the most recent data available in literature as well as expert opinions, was recently published outlining a wide array of challenges. The Consensus highlights the need to inform patients about expected rates of mechanical failure, erosion and infection which would result in a re-operation rates ranging from 14.8% to 44.8%. According to this publication, radiated patients are at high risk for increased adverse outcomes and complications, such as cuff erosion as well as re-operation, and should be informed about that. This same Consensus Conference have also outlined that currently the means to report the outcomes post-AUS are variable and need to become standardized.

The affiliation of the authors, the limitations of our observational study and the source of the data are clearly stated in the paper. Despite these limitations, the complication rate observed after AUS implantation should not be overlooked. In the absence of national clinical registries in Brazil, we understand that this study, based on an administrative data collection, is a source of “real-world” healthcare data on a large population of unselected patients. Although AUS implants are recommended as the gold-standard treatment for severe persistent urinary incontinence after prostatectomy, there are still several challenges regarding its indications, management, and follow-up to be overcome and discussed
